# Night Medical Service

**Published:** 1880-05

**Authors:** 


### NIGHT MEDICAL SERVICE.

A NIGHT medical service is now in operation in Paris, and in several of the large cities of Europe. It consists substantially in registering the names and addresses of those physicians, who are willing to do night service at the Police stations, so that strangers or those who are without a family physician, or those who wish to obtain a medical man in an emergency, can go at once to a station and be accompanied by an officer to the residence of the physician. The officer then goes with the Doctor to the residence of the patient and also escorts him on his return, and subsequently presents the bill, which, if the patient is unable to pay or cannot be found, is returned to the city, to be paid by the government, the fees being regulated by law.

